# Study protocol: Improving patient choice in treating low back pain (IMPACT - LBP): A randomised controlled trial of a decision support package for use in physical therapy

**DOI:** 10.1186/1471-2474-12-52

**Published:** 2011-02-25

**Authors:** Shilpa Patel, Sally Brown, Tim Friede, Frances Griffiths, Joanne Lord, Anne Ngunjiri, Jill Thistlethwaite, Colin Tysall, Mark Woolvine, Martin Underwood

**Affiliations:** 1 University of Warwick, Clinical Trials Unit, Warwick Medical School, Gibbet Hill Road, Coventry, CV4 7AL, UK; 2 Universities/User Teaching and Research Action Partnership (UNTRAP), Institute of Health School of Health and Social Studies, University of Warwick, Coventry, CV4 7AL, UK; 3 University Medical Centre Göttingen, Department of Medical Statistics, Humboldtallee 32, D-37073 Göttingen, Germany; 4 University of Warwick, Warwick Medical School, Gibbet Hill Road, Coventry, CV4 7AL, UK; 5 Brunel University, Health Economics Research Group, Middlesex, UB8 3PH, UK; 6 University of Warwick, Institute of Clinical Education, Warwick Medical School, Gibbet Hill Road, Coventry, CV4 7AL, UK; 7 NHS Coventry Community Physiotherapy, Coventry & Warwickshire Hospital, Stoney Stanton Road, CV1 4FH, UK

## Abstract

**Background:**

Low back pain is a common and costly condition. There are several treatment options for people suffering from back pain, but there are few data on how to improve patients' treatment choices. This study will test the effects of a decision support package (DSP), designed to help patients seeking care for back pain to make better, more informed choices about their treatment within a physiotherapy department. The package will be designed to assist both therapist and patient.

**Methods/Design:**

Firstly, in collaboration with physiotherapists, patients and experts in the field of decision support and decision aids, we will develop the DSP. The work will include: a literature and evidence review; secondary analysis of existing qualitative data; exploration of patients' perspectives through focus groups and exploration of experts' perspectives using a nominal group technique and a Delphi study.

Secondly, we will carry out a pilot single centre randomised controlled trial within NHS Coventry Community Physiotherapy. We will randomise physiotherapists to receive either training for the DSP or not. We will randomly allocate patients seeking treatment for non specific low back pain to either a physiotherapist trained in decision support or to receive usual care. Our primary outcome measure will be patient satisfaction with treatment at three month follow-up. We will also estimate the cost-effectiveness of the intervention, and assess the value of conducting further research.

**Discussion:**

Informed shared decision-making should be an important part of any clinical consultation, particularly when there are several treatments, which potentially have moderate effects. The results of this pilot will help us determine the benefits of improving the decision-making process in clinical practice on patient satisfaction.

**Trial registration:**

Current Controlled Trials ISRCTN46035546

## Background

Low back pain (LBP) is a common, disabling and expensive disorder. In 1998 treating this condition cost the National Health Service (NHS) £1,632M [1]. The 2009 National Institute for Health and Clinical Excellence (NICE) guidelines for early management of persistent non-specific LBP [2] recommend that clinicians consider offering patients with chronic LBP a course of one of a range of different therapies (acupuncture, exercise, manual therapy), depending on the patient's preference. These therapies have modest average benefits at a modest cost. The clinical effects and the costs of these different treatments are of a similar magnitude, so there is no clear basis for preferring any one of these treatments for use in the NHS. It is, however, possible that improved matching of patients to treatments will produce a greater overall positive effect. An understanding of the factors that inform patient choice and decision-making might help patients to make the best treatment decision for themselves. Having the opportunity to participate in such decision-making may also be associated with better health outcomes [3,4].

A patient-centred approach, using shared decision-making (SDM), involves the patient and healthcare professional discussing treatment options and interacting to agree on a management plan [5,6]. This approach is widely advocated but its use in practice is challenging [7]. SDM involves taking patient expectations, preferences, concerns, ideas and values, plus the practitioner's values and experiences into account. In contrast, informed decision-making involves presenting patients with relevant information and options without the healthcare professional expressing a preference. This 'informed model' gives patients autonomy but fails to take into account the shared interaction [8]. The patient-practitioner interaction model represents 'informed shared decision-making' (ISDM) and is the model that will be used to design a decision support package (DSP) for LBP patients. The quality of patient-practitioner interactions may be crucially important in improving back pain outcomes [9].

Coaching to support patients' making decisions is effective in aiding patients' knowledge, information recall, and participation in decision-making [10]. Decision aids are 'interventions designed to help people make specific and deliberate choices among options by providing information about the options and outcomes relevant to a person's health status' [10]. These aids provide information to facilitate patients' involvement in the decision-making process. Patient expectations and preferences also play a role in decision-making and may affect outcomes. Patients with a higher expectation of recovery have reported higher functional improvement [11]. Within randomised controlled trials (RCTs) in general, those randomised to their preferred treatment gain more benefit [12]. However, evidence from RCTs of LBP is equivocal: one RCT of LBP found that those who expected better outcomes with treatment gained more benefit than those who did not; however another trial failed to show that expectations affected outcome [13,14]. Patient preferences may also affect treatment adherence [15].

Patients report they would like to play an active role in decisions concerning their health [16]. Many patients also want a patient-centred approach to decision-making incorporating communication, partnership and health promotion [17]. However it is important to remember that while not all patients want to be involved in decision-making all of the time, they should be given choice about the depth of involvement. Clinicians do not always involve patients in decision-making; possibly because they feel ill trained to do so [18]. Despite this, general practitioners (GPs) have positive views about SDM and can gain the skills required to implement SDM in practice [19,20].

The aim of this pilot RCT is to develop and test the effect of a DSP on patient satisfaction. This package will comprise material to assist both the physiotherapist and patient during the informed shared decision-making process. Overall, the DSP will be designed to help patients seeking care for back pain, make informed choices, about their treatment which are the best for them.

## Methods/Design

This pilot has been funded by the National Institute of Health Research - Research for Patient Benefit Programme (Ref: PB-PG-0808-17039). Ethical approval has been granted by Warwickshire Research Ethics Committee (Ref: 10/H1211/2).

### Intervention design

To inform the design of the DSP, we will conduct a series of exploratory studies (described in more detail below). This work will be carried out in collaboration with physiotherapists, patients, and experts in the field of decision support and decision aids. At this time it is difficult to predict the exact nature of the DSP - this will depend on the findings from our exploratory work. However, we anticipate that there will be: an information package that summarises the nature of each intervention; a rationale for the interventions, questions that assist the patient in reviewing their current health status and the value they place on possible future scenarios, and what is known about the effectiveness of the interventions.

In a recent Cochrane review of decision aids for people facing health treatment or screening decisions, the included studies used a variety of decision aids for a range of conditions. A sample of some of the decision aids included were cancer treatments and screening, back surgery, hormone replacement therapy and osteoporosis treatments. The decision aids varied in formats including video tapes, computer based interactive programmes, audio material and information booklets [21]. Some decision aids were available in a combination of formats giving patients options. Our DSP will be designed to enable easy implementation into routine NHS care. This will be suitable for use by both patients and therapists. We anticipate that once this package has been developed, we will devise a formal training programme to deliver to physiotherapists to improve their knowledge of the treatment options and their skills in ISDM. We will integrate patients' and therapists' views on the content and format of our decision support package. We will subsequently publish a paper describing the intervention in detail.

In the early phase, we will ask physiotherapists within the Coventry Community Physiotherapy team to test the intervention in an uncontrolled manner, allowing us to test practicality and feasibility in the clinical environment. We will assess this by direct observation of the consultation process and through focus groups with patients and physiotherapists. This will allow us to optimise the intervention before starting the pilot RCT.

The section below describes the exploratory work to be undertaken to inform the intervention design.

#### A. Literature & evidence review

We will review relevant new trial data published since the 2009 National Institute for Health and Clinical Excellence (NICE) guidelines on low back pain [2]. This will allow us to identify any new data that, if available to the guideline development group, may have led to a change of recommendations. We will only include RCTs of therapist delivered interventions with a sample size >349. This is the sample size beyond which an RCT can detect a standardised effect size of 0.3 or less with 80% power and 5% significance if there are two groups split evenly [22]. It is such large trials that might, in the future, lead to changes in guidance. The information about the treatments and evidence in our DSP will be based on this NICE guidance and subsequent trials identified from this review.

We will systematically seek studies of decision aids for treatments for benign disorders, such as tennis elbow or depression, with multiple moderately effective treatment options. We will extract data about the components of these interventions and the mechanisms by which they are thought to act. We will use this data to inform the design of our decision aid.

We will also systematically identify qualitative studies of people with chronic back pain that include accounts of why they chose different therapies. This will provide us with some understanding of the reasoning behind treatment choice within patients with back pain.

#### B. Secondary Analysis

We have interview data from people living with back pain undertaken with individuals in both the intervention and control arm of a clinical trial evaluating a group cognitive behavioural approach for patients with low back pain in primary care (Back Skills Training Trial - BeST) [23]. Interviews were undertaken at 2-4 months after the intervention (or baseline assessment) and 12 months later. The interviews include exploration of the experience of living with back pain including the use of interventions beyond that being evaluated in the trial. We will re-analyse these data to identify influences on treatment decisions and use this data in our decision aid planning and development.

#### C. Patients' perspectives

Concurrently we will develop a questionnaire to measure current patient preferences for treatment. We will approach around 100 people seeking treatment from the physiotherapy department for LBP. Those interested will be asked to complete the questionnaire and subsequently participate in a focus group. Interested respondents will provide a sampling frame for our focus groups. Our purposive sampling willbe informed by age, gender, duration & troublesomeness of LBP, treatment decisions and treatment preferences. We will run two focus groups to develop a broad understanding of the factors that inform choice and decision-making among patients with back pain in which we will explore: how patients make decisions in general (i.e. decision style), how they make decisions and choices about treatments for their back pain, what information patients would like to help them make more informed choices, and what patients think of the existing material on offer to them.

Qualitative data will be analysed using the framework method to identify key concepts and themes [24].

#### D. Experts' perspectives

To develop an understanding of experts' views and experiences of informed shared decision-making for LBP treatments we have selected two broad areas of experts, physiotherapists and patient experts. By engaging these two groups we hope to generate a range of experiences which will feed into our intervention design.

We will recruit physiotherapists, who regularly provide first contact care for low back pain from a neighbouring primary care trust, to a nominal group - a formal face to face consensus method [25]. This is a structured approach to data collection from a group who have insight into a specific topic area. This method allows ideas to be generated as well as solutions to be achieved. Participants are initially asked to generate ideas, which the group later discuss and rank in order of importance. We have selected this method as it encourages participation from everyone and as a result we hope it will lead to good quality ideas and solutions.

Concurrently, we will also conduct a Delphi study with patient group leaders, expert patients and advocacy group activists. We will work with local and national organisations to identify expert patients who have experience of managing back pain. We have selected the Delphi method as it is a practical method of obtaining opinions from a range of experts nationally. We will develop a set of questions to send to the experts; the responses will then be collated and fed back to the participants for further rounds of questions. Rounds will continue until consensus is achieved.

The results of the reviews and secondary analyses together with the focus groups, nominal group technique and the Delphi study will feed into the intervention design. We will use the overall consensus of opinion to help inform how we develop, and what we include, in our decision support package. It is not possible at the time of writing to pre-judge the content and format of the decision support package. As part of the design process for the package we will invite comments from key opinion leaders in back pain research nationally and internationally to ensure that no key content has been omitted.

### Pilot RCT design

This study is a single centre randomised controlled trial taking place in the physiotherapy service at NHS Coventry Community Physiotherapy. The physiotherapy service sees around 300 patients as new back pain referrals a month. The service uses a paper free referral system, when patients are advised by their GP to attend the physiotherapy service, it is the patient's responsibility to call the booking team and arrange an appointment.

Physiotherapists will be randomised to either receiving training for the DSP or not. Subsequently patients will be randomised to either a trained or a standard physiotherapist.

#### Study population and eligibility criteria

One hundred and fifty participants seeking treatment from the physiotherapy service at NHS Coventry Community Physiotherapy will be recruited. We will include patients who have been advised by their GP to attend physiotherapy. GPs are asked to refer those patients presenting with LBP who do not return to normal activities after 3-4 weeks. Earlier referrals can be made for patients who are not coping with their pain e.g. the pain has a significant effect upon daily living or they present with poor prognosis which the GP is unable to address. In all cases the GP would provide patients with the Back Pain Access number. The patient is then free to ring and directly book a 45 minute assessment appointment. GPs are asked to exclude patients if they have signs of red flags, acute LBP of less than 3-4 weeks, those already under secondary care, had recent lumbar spine surgery, LBP as part of a presentation of multiple body pains e.g. fibromyalgia, thoracic or cervical pain and unstable psychiatric illness.

In this pilot RCT patients will be included if they are seeking treatment for non specific LBP, they are aged ≥18 years and have fluent spoken and written English. We will exclude patients with severe psychiatric or personality disorders, those with a terminal or critical illness and patients with possible serious spinal pathology (e.g. tumour, infection or fracture).

#### Recruitment procedure

**Figure 1:**
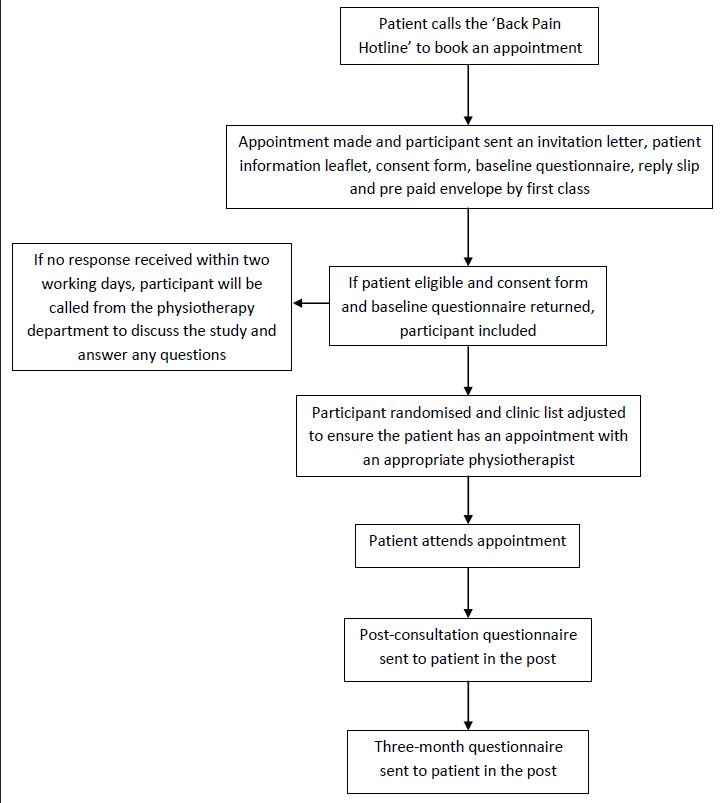
Pilot randomised controlled trial flow chart

Following advice from their GP, the patient is given information on how to get in touch with the booking service to make an appointment (see figure 1). Those who do so will be sent an invitation to join the study. Interested participants will return the consent form and completed baseline questionnaire to the research team. Patients who meet the inclusion criteria, complete the baseline questionnaire and consent to the trial will be included. They will be randomised to either a DSP trained physiotherapist or usual physiotherapy care. All participants will attend a 45 minute one-to-one assessment where they will be informed of the treatments on offer and have the opportunity to discuss these.

#### Randomisation

We will randomise half of the 14 (five whole time equivalents) physiotherapists to deliver the intervention. Patients will then be randomised to physiotherapists. Once the consent form and baseline questionnaire are received, the physiotherapy department will call the Warwick Clinical Trials Unit randomisation service to randomise the patient to either the control or intervention arm. Clinic lists will be adjusted to ensure participants have appointments with the allocated therapists: control or intervention.

#### Outcome assessment

Our package of outcome measures is based on those we have used successfully in two previous large scale community based trials of LBP treatments and are in line with international recommendations [26,27].

#### a. Primary outcome measure

We will use satisfaction with treatment at three months using a five-point Likert Scale (very satisfied to very dissatisfied) as our primary outcome. It is an amended version of the standardised and recommended single-item question [28]. Since a study of this size is unlikely to show a change in clinical outcomes, satisfaction with treatment is a useful outcome measure that is likely to be more sensitive to change than clinical outcomes. If, in this pilot, we can generate some evidence that we can improve satisfaction, we will have justification for proceeding to a trial to test the impact on clinical outcomes.

#### b. Secondary outcome measures

• Roland Morris Disability Questionnaire (RMDQ) [29] - The most commonly used measure of LBP-related disability in primary care trials.

• Modified Von Korff [30] - A measure of LBP & disability over the preceding month.

• SF-12 [31] - A generic measure of health-related quality of life.

• EuroQol [32] - A generic measure of health utility that is designed for use in RCTs.

• Hospital Anxiety and Depression Scale (HADS) [33] - An established and validated self rating instrument for anxiety and depression.

• Pain Self-Efficacy Questionnaire (PSEQ) [34] - An established measure self-efficacy for people with chronic pain.

• Fear Avoidance Beliefs Questionnaire (FABQ) [35] - The physical sub-scale of FABQ measures attitude to movement in back pain.

• Attendance - we will measure attendance from the physiotherapy department's records.

• Use of health services and costs

#### Immediate follow-up

The immediate follow-up assessment will focus on satisfaction with the decision-making process. We will use the 'Satisfaction with Decision Scale' [36]. This is a tool used to measure satisfaction with a health care decision. The scale has been adapted and applied to other conditions [37]. We will post this questionnaire to participants and ask them to return it by post to the study team.

#### Three-month follow-up

For this pilot RCT we will do a single follow-up after three months, when treatment will be complete and maximum benefit from treatment is expected. In addition to measuring satisfaction and repeating baseline measures we will collect data on health service use and health transition [38]. We will send follow-up questionnaires by post, three months after randomisation. We will send postal reminders after two and four weeks, and if necessary, we will collect a minimum data set by phone from non-responders.

#### Sample size

In the Back Skills Training (BeST) trial, satisfaction with treatment was measured using a five-point Likert Scale (very satisfied to very dissatisfied) [23]. In this trial the difference in satisfaction with treatment (a combination of 'somewhat satisfied' and 'very satisfied') at three months was 23.6% compared to 54.3% for control and treatment arm, respectively. Assuming satisfaction is 50% in the control group (similar to the treatment arm in BeST), then to show a similar additional improvement to 75% with 80% power at 5% significance level we need data on 58 participants in each group. We have not inflated the sample size to allow a design effect for clustering by therapist. In our two previous trials of LBP treatments, clustering effects by therapist were insignificant [26,27]. Thus, allowing for a 20% loss to follow up, we need to recruit a minimum of 150 participants.

#### Data analysis

Descriptive summary statistics for demographic and baseline data will be provided. The dichotomised responses on the five point Likert scale to the treatment satisfaction question will be analysed by means of a generalised linear mixed model with logit link, fixed effects for intervention and baseline pain severity, and random physiotherapist effects. The estimated intervention effect (odds ratio) will be reported with 95% confidence interval and p-value testing the null hypothesis of no intervention effect. Continuous outcomes will be reported as difference in change from baseline with its 95% confidence interval.

#### Health economics

We will estimate the mean cost of the intervention and other back pain-related NHS services and participants' out of pocket expenses over the three-month study period. We will collect information on the cost of training for the physiotherapists in the DSP group, including the cost of the tutors' time to prepare and deliver the training, the cost of participants' time, travel costs and materials.

Therapy cost will be collected from PCT records. The three-month follow-up questionnaire will include questions about use of medication (prescribed and over-the-counter), GP attendances, inpatient stays, outpatient consultations, and visits to other therapists (NHS and private). Healthcare cost will be estimated using unit costs from the NHS tariff and standard references texts [39].

The 'within-trial' difference in mean costs and the difference in mean quality adjusted life years (QALYs) will be estimated with 95% confidence intervals, allowing for the effects of treatment, baseline pain severity, and treating physiotherapist. These estimates will help us to assess the potential benefits of conducting a definitive trial and economic evaluation. For example, if the DSP is associated with greater healthcare costs and no apparent improvement in health outcomes over this initial three-month period, it is unlikely that further research would be worthwhile, particularly if there is also no clear evidence of a strong patient preference for use of the DSP. However, if cost savings and/or health improvements are observed, it is possible that DSP may be a cost-effective intervention and further research would be warranted.

## Competing interests

Martin Underwood was chair of the guideline development group for the NICE Low back pain guidelines. Joanne Lord was employed by NICE at the time of the development of the NICE Low Back Pain Guidelines and advised the guideline development group on economic analysis. All other authors declare that they have no competing interests.

## Authors' contributions

All authors have read and approved the final manuscript.

SB - has contributed to the design of the study and has commented on draft versions of this manuscript. TF - has contributed to the design of the study and is overseeing the quantitative analyses. TF has commented on draft versions of this manuscript. FG - has contributed to the design of the study and is overseeing the qualitative analyses. FG has commented on draft versions of this manuscript. JL - has contributed to the design of the study and is overseeing the economic analysis. AN - has commented on draft versions of this manuscript. SP - has substantially contributed to the conception and design of the study. SP has drafted this manuscript. CT - has contributed to the design of the study and has commented on draft versions of this manuscript. JT - has contributed to the design of the study and is overseeing the intervention design. JT has commented on draft versions of this manuscript. MW - has contributed to the design of the study. MU - is the principal investigator. He has substantially contributed to the conception and design of the study and obtaining the funding. MU has critically commented on draft versions of the manuscript.

## Pre-publication history

The pre-publication history for this paper can be accessed here:

http://www.biomedcentral.com/1471-2474/12/52/prepub
