# Correction: study protocol: improving patient choice in treating Low back pain (IMPACT - LBP): a randomised controlled trial of a decision support package for use in physical therapy

**DOI:** 10.1186/1471-2474-14-158

**Published:** 2013-05-03

**Authors:** Shilpa Patel, Sally Brown, Tim Friede, Frances Griffiths, Joanne Lord, Anne Ngunjiri, Jill Thistlethwaite, Colin Tysall, Mark Woolvine, Martin Underwood

**Affiliations:** 1 University of Warwick, Clinical Trials Unit, Warwick Medical School, Gibbet Hill Road, Coventry CV4 7AL, UK; 2 Universities/User Teaching and Research Action Partnership (UNTRAP), Institute of Health School of Health and Social Studies, University of Warwick, Coventry CV4 7AL, UK; 3 Department of Medical Statistics, University Medical Centre Göttingen, Humboldtallee 32, Göttingen D-37073, Germany; 4 University of Warwick, Warwick Medical School, Gibbet Hill Road, Coventry CV4 7AL, UK; 5 Brunel University, Health Economics Research Group, London, UK; 6 University of Warwick, Institute of Clinical Education, Warwick Medical School, Gibbet Hill Road, Coventry CV4 7AL, UK; 7 NHS Coventry Community Physiotherapy, Coventry & Warwickshire Hospital, Stoney Stanton Road, Coventry CV1 4FH, UK

## Summary of changes

After publication of this protocol a change in study design was needed [1]. Due to changes in the service configuration in the host physiotherapy department individual randomisation as originally planned could not be implemented. It was necessary to change to cluster randomisation with the unit of randomisation being the treating physiotherapist. Potential participants are given outpatient appointments by booking staff unaware of the physiotherapist’s randomisation. Trial recruitment is also done blind to physiotherapist allocation. In this manner we have ensured allocation concealment prior to participants joining the study. Cluster randomised trials need to inflate their sample size to account for clustering. Typically primary care trials use an intra-cluster correlation coefficient (ICC) of 0.05 in this calculation [2]. Our past experience is that clustering effects by therapist in trials of this nature may be very small [3]. To account for this we developed a provisional revised sample size using an ICC of 0.05 and did an interim analysis of pooled data, just for ICC of the primary outcome, after the first 40 participants had completed the three month follow-up questionnaire. The ICC was close to zero, suggesting that using an ICC of 0.05 was too conservative. We therefore assumed an ICC of 0.01 to estimate the design effect due to clustering. Based on an average cluster size of nine this results in a revised final sample size of 158.

Further details are provided in our correction article.

Trial registration

Current Controlled Trials ISRCTN46035546

## Full details

We initially applied for a substantial amendment on the 5^th^ of September 2011 to change the study design from individually randomised to cluster randomisation of patients by physiotherapists. Patients would therefore be allocated to the trial arm the consulting physiotherapist had been randomised to. As we changed the trial design the sample size was also adjusted to allow for clustering effects.

The original sample size was based on an 80% power and a two-sided type I error rate at 5%. Thus, 58 participants were required in each treatment arm. Allowing for a 20% loss to follow-up, the original total sample size was 150. In the change of the design, the sample size was adjusted to account for potential clustering effects. There were 11 clusters (physiotherapists) and assuming that the cluster size was between 21 and 22 and the intra-cluster correlation coefficient (ICC) was 0.05, a total between 232 and 238 participants was needed. Allowing for a 20% loss to follow-up, the total number of participants needed was between 286 and 308 [2]. The original power (80%) and significance level (0.05) remained the same.

Due to the substantial increase in sample size it was challenging to recruit to target. As this was a concern we planned a blinded sample size review (Friede and Kieser, 2006) to estimate the ICC due to clustering effects within this trial. Clustering effect by therapist tends to be smaller than 0.05 [3]. The analysis was done blind to treatment allocation and the data were frozen on 23 March 2012. By that date 40 participants had completed the 3-month follow-up questionnaires. Only two variables were extracted, the 5-point Likert item “Satisfaction with Treatment” (the primary endpoint of this trial) and the unique physiotherapist identifier number. The five levels of the Likert item ranges from “very satisfied” to “very dissatisfied” with a neutral option in between the positive and negative responses. The responses are dichotomised into “satisfaction” and “non-satisfaction”. The responses “very satisfied” and “somewhat satisfied” are classified as “satisfaction”. Whereas, the responses “neither satisfied nor dissatisfied”, “somewhat dissatisfied”, and “very dissatisfied” are categorised as “non-satisfaction”. There were 14 clusters (physiotherapists) and the cluster size ranges from 1 to 6. The method used for the estimation of the ICC was the generalized linear mixed model with logit link in SAS (PROC GLIMMIX). The estimated ICC was close to 0. This suggests that the true value for the ICC is smaller than the value of 0.05 we used originally. Therefore, for a re-calculation of sample size, we assumed that the ICC is 0.01. Allowing for an average cluster size of nine, at three months, and an ICC of 0.01 the design effect from clustering is 1.08. Thus we need data on 126 (116*1.08) participants at three months. Allowing for 20% loss to follow-up the total number of participants needed is 158 (i.e. 79 per arm). As this was a blinded sample size review, the p-value will not be adjusted in the final analyses [4].

The statistical analysis plan (SAP) was revised to reflect the change of the trial’s design, the revised sample size and the blinded sample size review. The estimated ICC from the blinded sample size review is also included in the SAP. In the original plan, the dichotomised 5-point Likert primary outcome (“Satisfaction with Treatment”) will be analysed by generalised linear mixed model with logit link. The study arm and baseline pain severity will form the fixed effects and the clusters will form the random effects. In the revised SAP, the generalised linear mixed model with logit link method is retained but with an additional fixed effect, namely, the physiotherapists’ years of experience (≤ 6 years vs. > 6 years). Also, the ICC will be estimated and reported with 95% confidence interval. The method is as described in Chakraborty *et al.* (2009) [5].

The revised protocol has ethical approval. The ethics committee did not deem a change in the sample size after the blinded interim review as requiring a substantial amendment.

## Pre-publication history

The pre-publication history for this paper can be accessed here:

http://www.biomedcentral.com/1471-2474/14/158/prepub
